# The Study of Steaming Durations and Temperatures on the Chemical Characterization, Neuroprotective, and Antioxidant Activities of *Panax notoginseng*

**DOI:** 10.1155/2022/3698518

**Published:** 2022-01-06

**Authors:** Didi Ma, Jue Wang, Guo Yin, Lijun Wang, Yibao Jin, Yang Huang, Kaishun Bi, Yi Lu, Tiejie Wang

**Affiliations:** ^1^School of Pharmacy, Shenyang Pharmaceutical University, Shenyang, Liaoning 110016, China; ^2^Shenzhen Institute for Drug Control, Shenzhen, Guangdong 518057, China; ^3^NMPA Key Laboratory for Quality Research and Evaluation of Traditional Chinese Medicine, Shenzhen Institute for Drug Control, Shenzhen, Guangdong 518057, China; ^4^Shenzhen Key Laboratory of Drug Quality Standard Research, Shenzhen Institute for Drug Control, Shenzhen, Guangdong 518057, China; ^5^Key Laboratory of Molecular Target & Clinical Pharmacology and the State Key Laboratory of Respiratory Disease, School of Pharmaceutical Sciences & the Fifth Affiliated Hospital, Guangzhou Medical University, Guangzhou, Guangdong 511436, China

## Abstract

*Panax notoginseng* (PN) is one of the most valuable traditional Chinese medicines and has extensive pharmacological effects. Recent studies demonstrated that PN exhibited pharmacological effect related to Alzheimer's disease (AD). However, whether steaming process can boost its anti-AD activity is still unexplored. To fill this gap, effects of steaming durations and temperatures on the chemical characterization, neuroprotective and antioxidant activities of PN were systematically investigated in this study. HPLC fingerprint coupled with quantitative analysis demonstrated striking conversion of original saponins to less polar ones with the increase in the steaming time and temperature. In the viewpoint of anti-AD activity on neuroprotective and antioxidant effects, several steamed PN samples (110°C-6/8/10 h, 120°C ‐4/6 h samples) displayed a significant increase both in cell viability and oxygen radical absorption capacity (ORAC) values compared with the no steamed one (*P* < 0.01 or *P* < 0.005). Steaming temperature had the greater impact on the change of chemical composition and anti-AD activity of PN. Moreover, the spectrum-effect relationship analysis revealed that the transformed saponins were partially responsible for the increased neuroprotective and antioxidant effects of steamed PN. Therefore, steamed PN could be used as a potential crude drug for prevention and treatment of AD.

## 1. Introduction

Alzheimer's disease (AD) is the leading cause of age-related dementia and a devastating neurodegenerative disorder worldwide [1]. In the absence of any effective preventive measures and therapeutic interventions, AD is, therefore, considered as a public health threat for our aging society. Mounting evidence supports that the accumulation of amyloid-*β* (A*β*) and oxidative stress are crucial pathogenesis of AD [2, 3]. In AD patients, a 40- or 42-amino-acid peptide (A*β*_1-40_ or A*β*_1-42_) called A*β* is raised in the brain [4]. The toxic soluble A*β* oligomers, intraneuronal A*β*, and extracellular amyloid plaques (consisting of aggregated A*β*) impair synaptic functions and trigger serial toxic pathways which ultimately results in neurodegeneration [5]. Besides, the brain is highly susceptible to oxidative damage [6]. Oxidative stress not only causes oxidative damage to neuronal macromolecules but also occurs early in the course of AD, which supports its important role in disease pathogenesis [7, 8]. Therefore, targeting damage induced by A*β* as well as oxidative stress in the brain is a promising therapeutic strategy to prevent and improve AD symptoms [9].

Traditional Chinese medicine (TCM) has accumulated thousands of years of experience in treating dementia [10]. It is usually viewed as more accessible and acceptable form of treatment due to multitargeted approach, synergistic effect, high selectivity, and low toxicity [11, 12]. Nowadays, two well-known AD therapeutic agents, huperzine A (HA) and galantamine, have been discovered and isolated from TCMs, indicating that TCM is a promising source of latent anti-AD drug [13]. *Panax notoginseng* (PN) or Sanqi, a highly valued TCM, which has been used for over four centuries [14]. It possesses various pharmacological activities, such as anticancer, antidiabetic, anti-inflammatory, and antioxidant [15]. Several studies have found that saponins, the major active ingredients of PN, exerted beneficial effects on AD [16, 17]. Li et al. [18] showed that notoginsenoside R_1_ had a neuroprotective effect on an APP/PS1 double-transgenic mouse model of AD by upregulating insulin degrading enzyme and suppressing A*β* accumulation. Liu et al. [19] reported that ginsenoside Rd treatment ameliorated cognitive function on A*β*_1-40_-induced AD model rats by anti-inflammation, antioxidation, and antiapoptotic. Therefore, PN is considered as one of the promising candidates with anti-AD potential [20]. However, it is traditionally used in two different forms: the raw and steamed forms. After steaming, plenty of transformed saponins (ginsenosides Rg3, Rk3, Rh4, and Rg5) converted from primary saponins of raw PN were observed [21]. The potential efficacy of transformed saponins on the treatment of AD has drawn significant attention. Zhang et al. [22] showed that ginsenoside Rg_3_ could prevent the cognitive impairment of AD rats by improving the mitochondrial dysfunction. Chu et al. [23] demonstrated that ginsenoside Rg_5_ improved learning and memory function in streptozotocin-induced AD rats through regulating the cholinergic system, attenuating A*β* deposition, and enhancing the expression of neurotrophic factors (BDNF, IGF-1). These studies have presented that some saponin ingredients of raw and steamed PN exhibited pharmacological effects related to AD, while few attempts have been conducted to analyze and compare the overall effects of raw and steamed PN on anti-AD activity.

Therefore, in this study, raw and steamed PN were selected to assess their anti-AD activities focusing on anti-A*β*_1-42_ and antioxidant effects. The raw PN was processed with different steaming temperatures and durations to produce different steamed PN samples. Thereafter, the influence of different steaming parameters on the change in chemical composition and anti-AD activity was systematically evaluated. The steaming condition of PN for the anti-AD test was optimized by the aforementioned results. In addition, the spectrum-effect relationship between chemical data and anti-AD activity was used to explore the potential anti-AD constituents in raw and steamed PN.

## 2. Materials and Methods

### 2.1. Chemicals and Reagents

The reference standards of notoginsenoside R_1_ and ginsenosides Rg_1_, Re, Rb_1_, Rg_2_, Rh_1_, Rd, 20*(S)*-Rg_3_, and 20(*R*)-Rg_3_ were purchased from Chengdu Must Bio-Technology Co., Ltd. (Chengdu, China). Ginsenosides Rk_3_, Rh_4_, and Rg_5_ were obtained from Chengdu Chroma-Biotechnology Co., Ltd. (Chengdu, China). HA was purchased from National Institutes for Food and Drug Control (Beijing, China). HPLC-grade methanol, acetonitrile, and dimethyl sulfoxide (DMSO) were provided by Merck Drugs & Biotechnology (Darmstadt, Germany). Ultrapure water was prepared by a Milli-Q system (Millipore, Bedford, USA). Fluorescein disodium and 3-(4,5-dimethyl-2-thiazolyl)-2,5-diphenyl-2H-tetrazolium bromide (MTT) were from Aladdin Industrial Corporation (Shanghai, China). PBS was purchased from Beyotime Biotechnology (Shanghai, China). A*β*_1-42_, 6-hydroxy-2,5,7,8-tetramethyl-2-carboxylic acid (Trolox) and 2,2′-Azobis (2-methylpropionamidine) dihydrochloride (AAPH) were bought from Sigma-Aldrich (St. Louis, USA). Analytical grade potassium dihydrogen phosphate and dipotassium hydrogen phosphate were acquired from Guangzhou Chemical Reagent Factory (Guangzhou, China) for preparing phosphate buffer. RPMI 1640 medium, fetal bovine serum (FBS), penicillin-streptomycin, and trypsin were purchased from Life Technologies Corporation (NY, USA).

### 2.2. Plant Material and Preparation of Extract

The raw PN roots aged 3 were collected from Wenshan TCMs market (Yunnan, China) and authenticated by Shuhong Wang from Shenzhen Institute for Drug Control. Dry roots were grounded into powder with a high-speed pulverizer and then filtered by 65 mesh sieves. To obtain steamed PN, above-mentioned powder was processed by an autoclave. Steaming parameters included different temperatures (105, 110, and 120°C) and different durations (2, 4, 6, 8, and 10 h) were set to optimize the processing method. Then, the steamed powders were dried at 60°C [24, 25]. In addition, the steamed samples were represented by different steaming parameters, such as 105°C-2 h.

The extracts of raw and steamed PN were prepared following previous extraction procedures [26] with minor modify. An amount of 0.6 g of each sample, in the powdered form and filtered through 65 mesh sieves, was transferred to a 150 mL conical flask with stopper adding 50 mL methanol-water (70:30, v/v) solution as a solvent. Then, ultrasonic extraction was performed for 40 min at room temperature. After cooling and compensating the weight loss during the extraction with methanol-water (70 : 30, v/v) solution, the extract was filtered through a 0.45 *μ*m membrane and stored at 4°C until HPLC/oxygen radical absorption capacity (ORAC) analysis. For the anti-A*β*_1-42_ study, each sample was extracted by the similar procedure mentioned above. Afterwards, all extracts were concentrated and lyophilized to avoid solvent. Before use, the dried extract was dissolved in RPMI 1640 medium and diluted to the desired concentration for the cell experiments.

### 2.3. Chemical Composition Assay

A Shimadzu LC-20A HPLC system equipped with an auto-sampler, a quaternary pump, and a DAD was applied. All separations were carried out on a GEMINI-NX C18 column (250 mm × 4.6 mm, 5 *μ*m, Phenomenex) maintained at 25°C. The mobile phase consisted of water (A) and acetonitrile (B) went through a gradient elution as 17.5%–21% (v/v) B for 20 min, 21%–26% B for 3 min, 26% B for 6 min, 26%–36% B for 5 min, 36%–50% B for 11 min, 50%–55% B for 2 min, 55% B for 5 min, 55%–100% B for 5 min, 100% B for 3 min, 100%–17.5% B for 5 min, and 17.5% B for 5 min. The injection volume and flow rate were set at 10 *μ*L and 1.0 mL/min, and the detection wavelength was fixed at 203 nm.

### 2.4. Anti-A*β* Peptide Assay

The PC12 cell, a cell lineage derived from a pheochromocytoma of rat adrenal medulla, is widely applied as experimental model for neuroprotection and neurodegeneration analyses, and A*β* is usually considered as the main toxic agent in AD [27, 28]. This study aimed to estimate the potential neuroprotective activity of raw and steamed PN samples by reducing the cytotoxicity of A*β*_1-42_ in PC12 cells. The PC12 cells (Shanghai Institute of Biochemistry and Cell Biology, Shanghai, China) were grown in RPMI 1640 medium containing 10% (v/v) FBS and 1% (v/v) penicillin-streptomycin agent at 37°C under 5% CO_2_ humidified atmosphere. To ensure the selected concentration of sample had no significant cytotoxicity, the MTT assay was applied to determine the cell viability. Typically, 100 *μ*L cells were seeded at the density of 2 × 10^4^ cells per well in 96-well plates and incubated overnight. PC12 cells were treated with several different concentrations (50, 100, and 200 *μ*g/mL) of extracts for 24 h. Thereafter, the medium in each well was removed and replaced by 20 *μ*L of MTT solution (5 mg/mL in PBS) for an additional 4 h. Finally, the MTT was aspirated and insoluble purple formazan crystals produced by live cells were solubilized in 150 *μ*L of DMSO. The plates were placed on a rocking shaker for 15 min and then measured at 570 nm with a reference wavelength of 630 nm by a Varioskan Flash multimode microplate reader (Thermo Fisher Scientific, Waltham, USA). Cells without drug exposure were used as vehicle control. The result was expressed as the percentage relative to that monitored in the vehicle control group. All experiments were carried out in triplicate and repeated duplicate.

To select an optimal concentration for A*β*_1-42_ treatment, cell viability was determined by the same procedure as above with minor modifications. PC12 cells were incubated with 1, 5, and 10 *μ*M A*β*_1–42_ for 24 h. Normal PC12 cells without A*β*_1–42_ treatment was used as the vehicle group.

Similarly, the potential neuroprotective activity of samples was evaluated by reducing the cytotoxicity of A*β*_1-42_ in PC12 cells. The PC12 cells were treated with A*β*_1-42_ and co-incubated with different extracts or HA for 24 h prior to measurement of cell viability. HA standard (10 *μ*M) was served as positive control. A*β*_1–42_ added to the treatment without the addition of samples as the model group. Normal PC12 cells without samples and A*β*_1–42_ treatment was used as vehicle control.

### 2.5. Antioxidant Assay

The ORAC assay was applied to estimate the antioxidant activity of samples. This assay reveals relatively dynamic information on radical chain-breaking capacity with peroxyl radicals, which is widely employed to determine the antioxidant capacity of the individual compound or complex mixture [29]. It assesses the effectiveness of an antioxidant with fluorescein as a probe by inhibiting the fluorescence decay that is induced by a peroxyl radical generator, AAPH, and provides a unique and complete procedure in which the inhibition time and inhibition degree are monitored as the reaction process from start to finish [30]. The ORAC assay was performed according to the modified method as described by Ou et al. [31]. Sodium fluorescein and AAPH were both dissolved in phosphate buffer (pH 7.4, 75 mM) at the final concentration of 0.789 *μ*M and 173 mM, respectively. An aliquot (25 *μ*L) of 300 *μ*g/mL extract and 100 *μ*L of sodium fluorescein were added into black-walled 96-well plate and incubated at 37°C for 10 min. Then, the reaction started when adding 75 *μ*L of AAPH solution. The fluorescence was collected every 2 min for 120 min at 485 nm excitation wavelength and 515 nm emission wavelength in a microplate reader. Trolox, a water-soluble analog of vitamin E, was used as a standard, and 75 mM phosphate buffer was used as a blank. The result was calculated as the relative area under the fluorescence decay curves between the blank and sample, expressed as micromoles of Trolox equivalent (TE) per gram (*μ*mol TE/g). Experiments were performed in triplicate.

### 2.6. Data Analysis and Statistics

Statistical analysis was performed using GraphPad Prism 8.0 statistical software (GraphPad Software, Inc., San Diego, CA, USA). Data were expressed as mean ± standard deviation (SD) and analyzed by one-way ANOVA followed by Tukey's multiple comparison, variables with *P* < 0.05 were considered statistically significant (^*∗*^^/#^*P* < 0.05, ^*∗∗*^^/##^*P* < 0.01 and ^*∗∗∗*^^/###^*P* < 0.001). The hierarchical cluster analysis heatmap was performed by Heml 1.0 (CUCKOO Workgroup, Wuhan, China). The partial least square was carried out on SIMCA-P 11.5 software (Umetrics, Umea, Sweden).

## 3. Results

### 3.1. Method Validation

To verify the applicability of quantitative analysis of selected components, the method was evaluated based on linearity, limit of detection (LOD), limit of quantification (LOQ), accuracy, precision, repeatability, and stability assay. The linearity of quantitative analysis was performed by determining a series of mixed standard solutions with varying concentrations. The calibration curve of each analyte was constructed by plotting the peak areas (y) against the concentrations (x, mg/mL). As shown in Supplementary Table 1, all the analytes showed excellent linearity (*r* > 0.999) within relatively wide ranges. The LODs and LOQs were examined by injecting serial diluted standard solutions and taking peaks with signal-to-noise rate of 3 and 10 as criteria, which were in the range of 0.34–1.43 *μ*g/mL and 0.85–5.38 *μ*g/mL, respectively. The accuracy was estimated by calculating the recovery with the standard addition method. The mean recovery of each compound ranged from 95.63% to 103.40% with an RSD value <3.25%. The precision was examined by analyzing the same sample solution with 6 consecutive runs; repeatability was assessed by analyzing 6 independent replicate extracts of one sample; stability was tested by analyzing the same sample extract at different time intervals (0, 4, 8, 12, 16, 20, and 24 h). The precision, repeatability, and stability were represented by RSD values of peak areas of 12 analytes, which were less than 0.94%, 2.57%, and 2.55%, respectively.

To ensure that the established method can be applied to HPLC fingerprint analysis, the validation was carried out with precision, repeatability, and stability assay. The results were represented by RSD values of relative retention times and relative peak areas of 19 characteristic peaks concerning the reference peak (peak 9) at the retention time of 42.3 min. The variation of the relative retention times and relative peak areas of the characteristic peaks did not exceed 0.29% and 1.93%, respectively.

### 3.2. Determination of Chemical Compositions in Extracts

**Figure 1:**
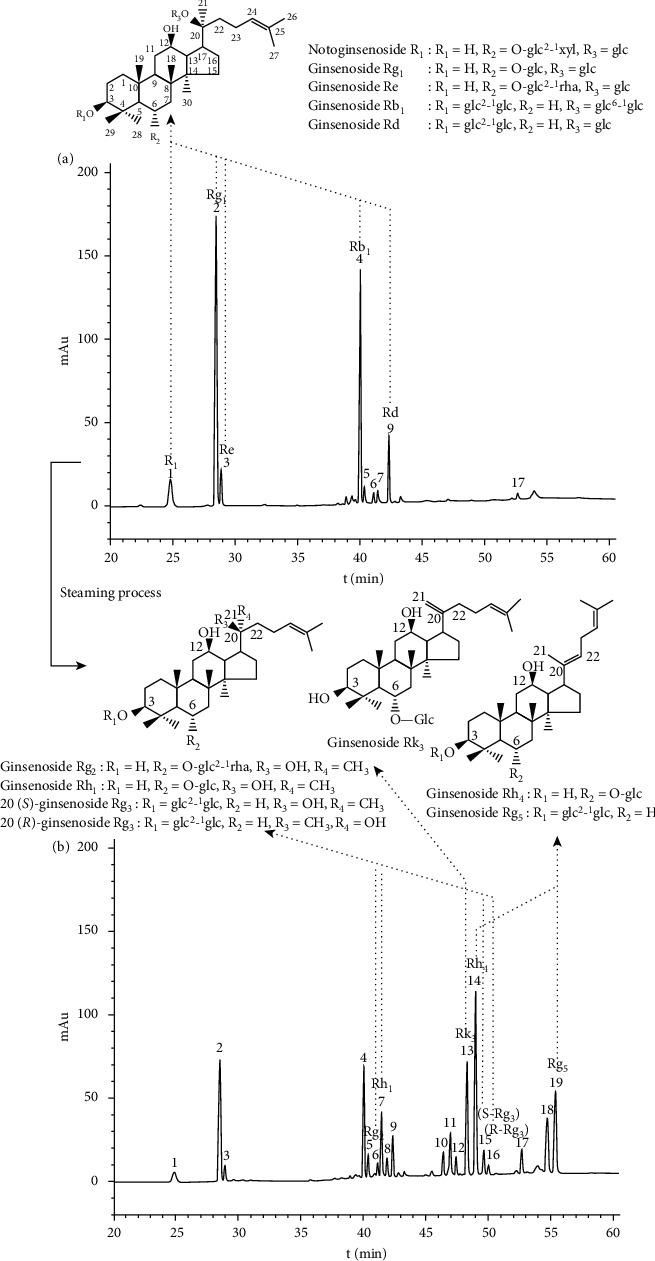
Typical chromatograms of raw (a) and 120°C-4 h steamed (b) *Panax notoginseng*.

**Figure 2:**
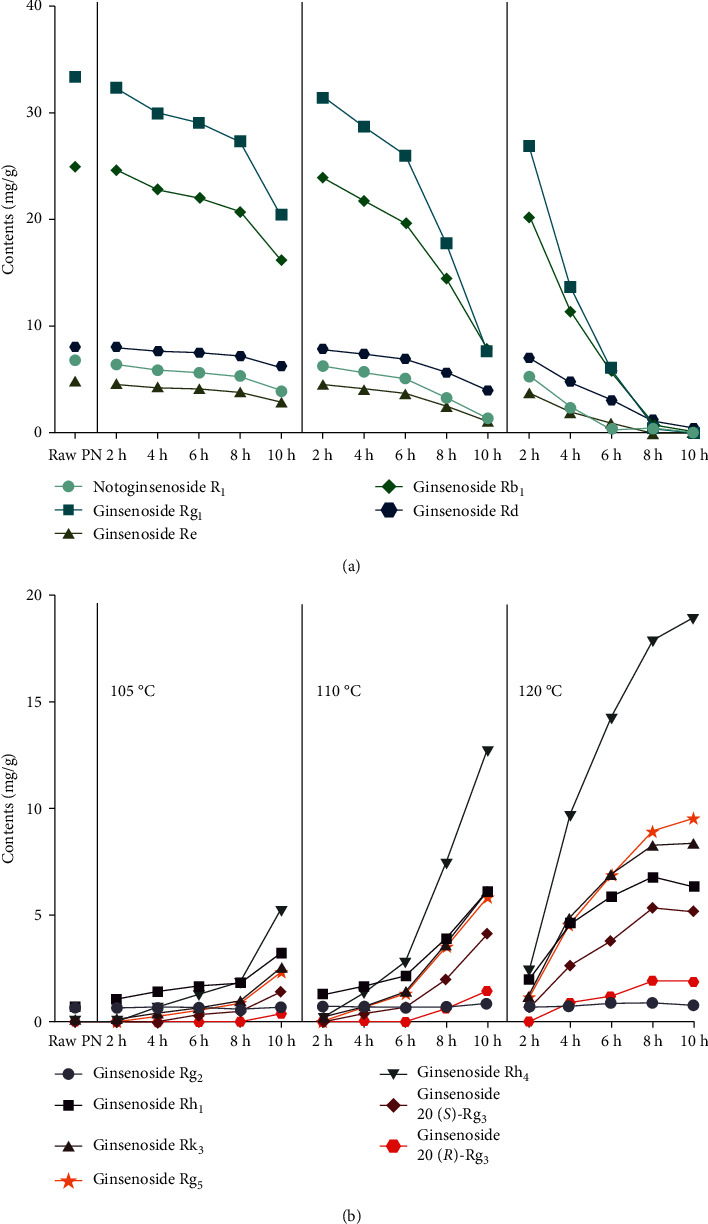
The degradation curves (a) and enhancement/formation curves (b) of saponins in *Panax notoginseng* (PN) during the steaming process.

Typical HPLC chromatograms of raw and steamed PN are shown in Figures 1(a) and 1(b), respectively, and 12 saponin constituents were identified by comparing the DAD spectrums (Data not shown), retention times with standard compounds, and spiking extracts with the standard substances further confirmed the identifications. The observed changes in individual constituents of different samples are shown in Figure 2. The contents of saponins varied during the steaming process. As shown in Figure 2(a), the original saponin (notoginsenoside R1 and ginsenosides Rg_1_, Rb_1_, Re, and Rd) contents substantially decreased by steaming treatment, particularly ginsenosides Rg_1_ and Rb_1_, ranged from 33.45 and 25.01 mg/g to undetectable, respectively. On the other hand, the transformed saponins (ginsenosides Rg_2_, Rh_1_, Rk_3_, Rh_4_, 20(*S*)-Rg_3_, 20(*R*)-Rg_3_, Rg_5_) were enhanced or formed during the steaming process (Figure 2(b)). Among them, the contents of ginsenosides Rk_3_, Rh_4_, 20(*S*)-Rg_3_, and Rg_5_ were undetectable by HPLC in the raw PN, and up to 8.38, 18.89, 5.16, and 9.54 mg/g for 120°C-10 h sample, respectively. During the steaming process, the extent of transformation increased slowly in the first 8 h at 105°C and first 6 h at 110°C; subsequently, the contents of the main degradation products were increasing rapidly. In the case of steaming at 120°C, the formation of transformed saponins was increased sharply from 2 h to 10 h. The extent of transformation of 120°C-6 h sample was higher than that of 105°C-10 h and 110°C-10 h samples. However, even steaming at 120°C for 10 h, the original saponins could be detected and the contents of transformed products were still increasing. By comparing the steaming conditions of PN, it was easy to find that the change of saponins was in a time-dependent and temperature-dependent manner in this study. Therefore, both steaming time and steaming temperature had the important role in changing the chemical compositions of PN. Compared with the steaming time, the steaming temperature was the more critical factor for the content change of saponins.

**Figure 3:**
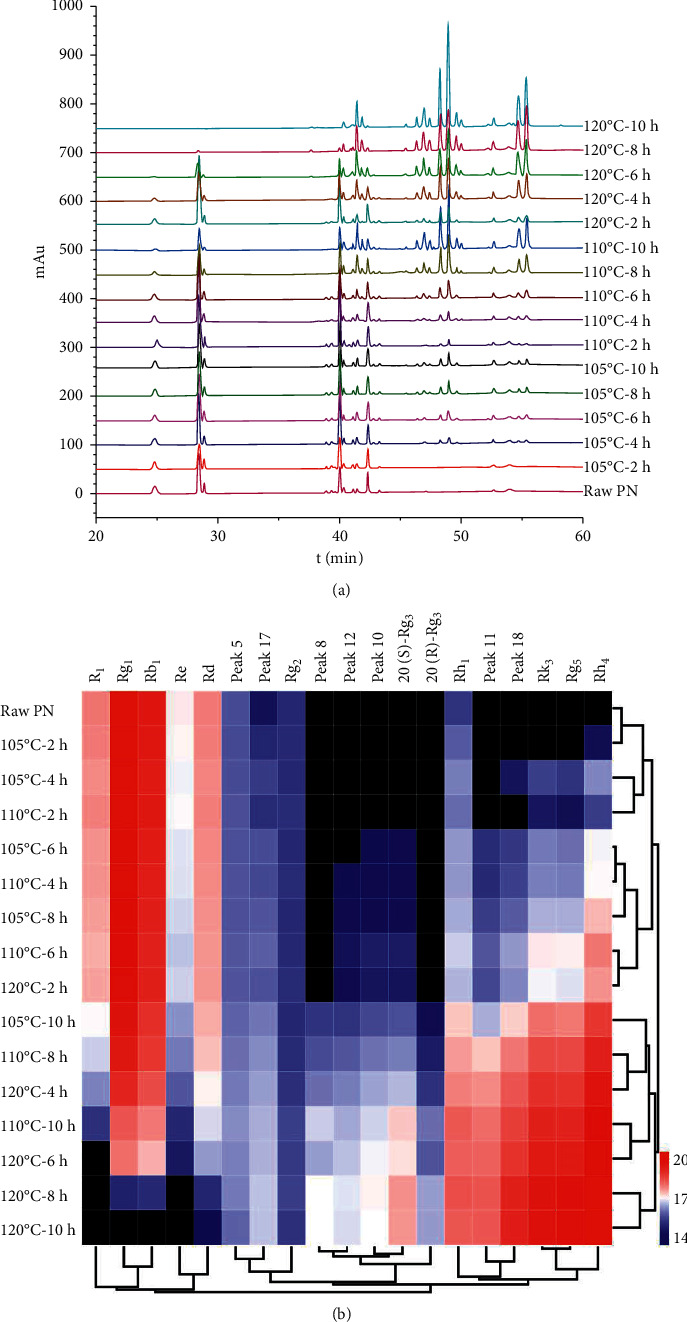
HPLC fingerprints (a) and hierarchical cluster analysis heatmap (b) of *Panax notoginseng* (PN) samples with different steaming processes. The steamed samples were represented by different steaming conditions.

HPLC fingerprints of PN samples were obtained and are displayed in Figure 3(a). A total of 19 peaks from consecutive peaks with good segregation were assigned as the characteristic peaks. To systematically and visually characterize the similarities and differences among samples, a hierarchical cluster analysis heatmap of peak areas of all the characteristic peaks was constructed (Figure 3(b)). The colour of the heatmap gave an overview of differences in values and the black represented zero. The result displayed that the chemical composition and content of samples were significantly different. In hierarchical cluster analysis, chromatographic peaks were horizontally clustered, and samples were vertically clustered. The result demonstrated that the characteristic peaks were well divided into 2 groups. Notoginsenoside R_1_ and ginsenosides Rg_1_, Rb_1_, Re, and Rd, the original main constituents in raw PN, belonged to the content-decreased group, and other components belonged to the content-increased group. Besides, all the samples were categorized into 4 clusters: cluster I contained raw PN and some steamed samples (105°C-2/4 h, 110°C-2 h samples) with similar chromatograms; cluster II comprised the materials (105°C-6/8 h, 110°C-2/4/6 h, 120°C-2 h samples) that were processed with mild treatment conditions; the samples (105°C-10 h, 110°C-8/10 h, 120°C-4/6 h samples) belong to cluster III were subjected to moderate steaming procedures; cluster IV consisted of others (120°C-8/10 h samples) with severe heat treatments. It was worth noting that the chemical profiles of steamed PN belong to cluster III and IV were significantly different from raw form.

The contents of saponins combined with hierarchical cluster analysis of chemical profiles was adopted for further analysis. For the samples of cluster I and cluster II, original saponins slightly converted to less polar compounds, and the total contents of confirmed converted compositions ranged from 1.37 (raw PN) to 3.39 (110°C-2 h sample in cluster I) and 8.93 mg/g (110°C-6 h sample in cluster II), respectively. For the samples of cluster III, the total contents of seven transformed saponins were markedly increased and up to 39.65 mg/g for 120°C-6 h sample. In the case of cluster IV, the total contents of transformed less polar degradation compounds were dramatically increased and reached 50.91 mg/g for 120°C-10 h sample. The result indicated that we could obtain more converted saponins with steaming conditions of cluster III and IV.

### 3.3. Neuroprotective Activity of Extracts against A*β*1-42

**Figure 4:**
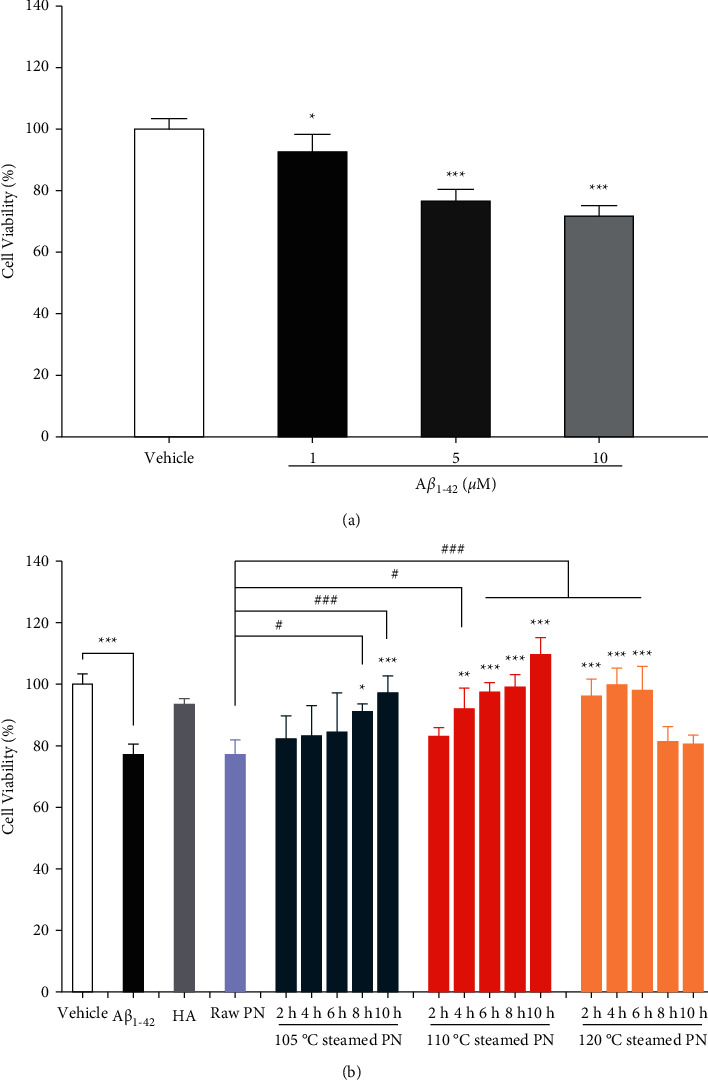
Protective effect of raw and steamed *Panax notoginseng* (PN) extracts suppressed A*β*_1-42_-induced cell viability loss in PC12 cells. (a) Cells were treated with A*β*_1-42_ at the indicated concentrations (1, 5, and 10 *μ*M) for 24 h and cell viabilities were assessed by MTT assay. (b) Cells were treated with A*β*_1-42_ (5 *μ*M) and co-incubated with raw or steamed PN extracts (100 *μ*g/mL) for 24 h and cell viabilities were assessed by MTT assay. Huperzine A (HA, 10 *μ*M) was served as positive control. ^*∗*^*P* < 0.05, ^*∗∗*^*P* < 0.01, ^*∗∗∗*^*P* < 0.005 versus vehicle control group or A*β*_1-42_-induced model group as indicated, ^*#*^*P* < 0.05, ^*##*^*P* < 0.01, ^*###*^*P* < 0.005 versus the raw PN treated group were considered statistically significant differences.

The cytotoxic effects of samples at different concentrations (50, 100, and 200 *μ*g/mL) were evaluated in PC12 cells by the MTT assay first. As shown in Supplementary Figure 1, all the samples had no considerable negative effect on the cell viability at the dose of 50 and 100 *μ*g/mL. However, the viability exhibited a significant decrease when treated with 120°C-10 h sample at 200 *μ*g/mL. The results demonstrated that all the samples on the inhibition of culture were not significant below 100 *μ*g/mL. Thus, 100 *μ*g/mL of extracts were subsequently used in the following experiments. Afterwards, we used A*β*_1-42_ to induce neuronal toxicity to explore the anti-A*β* property of PN. As shown in Figure 4(a), treatment of cells with different concentrations (1, 5, and 10 *μ*M) of A*β*_1-42_ for 24 h resulted in a significant decrease of the cell viability in a dose-dependent manner. Finally, 5 *μ*M of A*β*_1-42_ was chosen as the damage concentration due to a significant difference in viability compared with the vehicle control group.

Based on the results described above, the neuroprotective activity of raw and steamed PN extracts against A*β*_1-42_-induced cell damage were examined by the MTT assay. As shown in Figure 4(b), when PC12 cells were exposed to 5 *μ*M of A*β*_1-42_ without treatment of extracts, the cell response significantly decreased as compared with the vehicle control group. The viability of each PN treatment group, to varying extents, was higher than that of A*β*_1-42_ induced model group. However, there was no significantly effective effect of raw PN against A*β*_1-42_-induced toxicity. When raw material was processed at 105°C, the cell response enhanced as the steaming duration increased. After steaming for 8 h, the sample exhibited a slight neuroprotective effect, and the 10 h sample performed better. In the case of 110°C, the cell response presented similar trend with 105°C result and had significant improvement for the samples with steaming time ranged from 4 to 10 h. However, at 120°C, the viability increased first and then decreased with the increase of steaming time of PN. Processing for 2, 4, and 6 h samples could alleviate A*β*_1-42_-induced damage, 8 and 10 h samples had no neuroprotective effect. For the same steaming time, the anti-A*β*_1-42_ property of samples was in a temperature-dependent way in the first 6 h, the viability first increased and subsequently decreased with the steaming time of 8 and 10 h. The neuroprotective effect of 120°C-4 h sample was similar to 105°C-10 h and 110°C-8 h samples. The results also indicated that compared with the steaming time, the steaming temperature in the steaming process had the greater impact on the change of neuroprotective effect of PN. Besides, all the steamed samples with neuroprotective effects also displayed a significant increase in cell viability when compared with the raw PN treated group. Furthermore, the anti-A*β*_1-42_ activity of some steamed PN (105°C-10 h, 110°C-6/8/10 h, 120°C-2/4/6 h samples) was similar to that of HA (positive control). Based on the results, the steaming process improved the anti-A*β*_1-42_ property of PN.

### 3.4. Effect of Extracts on Antioxidant Activity

**Figure 5:**
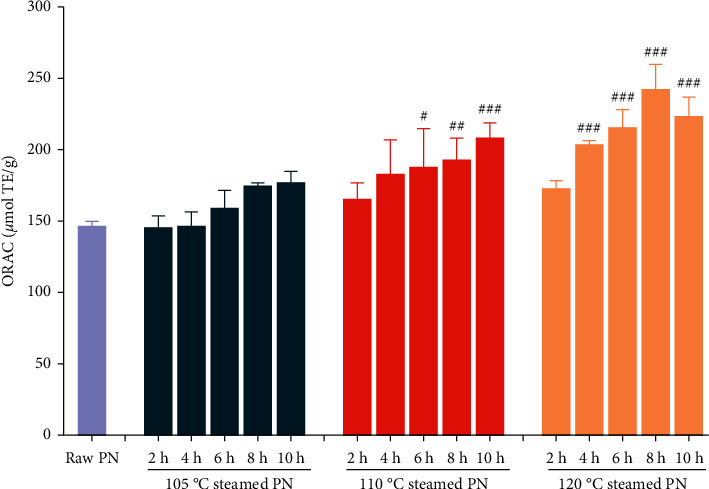
Oxygen radical absorbance capacity (ORAC) values of raw and steamed *Panax notoginseng* (PN) extracts. ^*∗*^*P* < 0.05, ^*∗∗*^*P* < 0.01, ^*∗∗∗*^*P* < 0.005 versus the raw PN treated group was considered statistically significant differences.

In this work, the ORAC method was applied to measure the antioxidant activity of PN extracts. As shown in Figure 5, raw PN exhibited the lowest antioxidant capacity among the samples. When the raw material was steamed at 105°C, there was no significant influence on the ORAC value even processing for 10 h. At 110°C, the antioxidant activity steadily increased and had significant improvement for the samples with longer steaming time than 6 h. In the case of 120°C, the ORAC values sharply increased during the first 8 h, and then decreased at 10 h. Steaming for 4–10 h samples had significantly greater ORAC values than no steamed one. While for the same steaming time, the ORAC value was in a temperature-dependent way. Like the neuroprotective effect, the antioxidant activity of 120°C-4 h sample is similar to the 105°C-10 h and 110°C-8 h samples. Therefore, steaming temperature was the more important factor on the change of antioxidant activity.

### 3.5. Spectrum-effect Relationship Analysis

**Figure 6:**
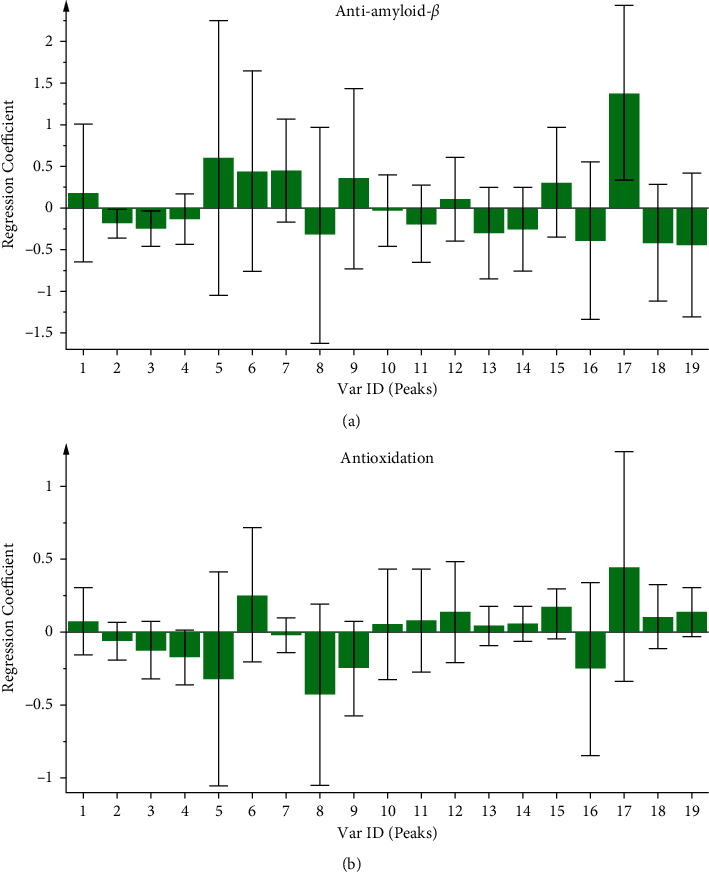
Regression coefficients of the anti-amyloid-*β* (a) and antioxidation (b) obtained with PLS.

Partial least square (PLS), a statistical procedure for extracting useful information from data, specifies a linear relationship between variables and has been widely applied to study the spectrum-effect relationship. In this work, it was employed to explore the main chromatographic peaks, which might contribute to the anti-AD activity of raw and steamed PN. Overtreatment reduced the efficacy of extracts; therefore, 120°C-8/10 h and 120°C-10 h samples were refused to establish anti-A*β*_1-42_ and antioxidant models, respectively. The established models had high explanatory power for anti-A*β*_1-42_ activity (*R*^2^ = 0.933) and antioxidant activity (*R*^2^ = 0.931). In the spectrum-efficacy model, the areas of 19 chromatographic peaks of samples were the predictor variables, and two indexes about anti-AD were the response variables. The relative influence of the predictor variable on the response variable was expressed by the regression coefficient. If the regression coefficient of one chromatographic peak was greater than zero, then it contained the anti-AD component. For the anti-A*β*_1-42_ model, there were 8 chromatographic peaks (peaks 1, 5, 6, 7, 9, 12, 15, and 17) with the inhibitory effect on A*β*_1-42_-induced cytotoxicity in PC12 cells (Figure 6(a)). For the antioxidant model, 11 chromatographic peaks with antioxidant activity were peaks 1, 6, 10, 11, 12, 13, 14, 15, 17, 18, and 19 (Figure 6(b)). Combined with the anti-A*β*_1-42_ and antioxidant results, the peaks 1, 6, 12, 15, and 17 were considered as the potential active ingredients that respond to anti-AD. Three of them were identified as notoginsenoside R_1_ (P1), ginsenosides Rg_2_ (P6), and 20(*S*)-Rg_3_ (P15) respectively. Among identified components, notoginsenoside R_1_ exclusively existed in the raw PN, and ginsenosides Rg_2_ (P6) and 20(*S*)-Rg_3_ (P15) considerably existed in the steamed form. Therefore, notoginsenoside R_1_ might be the main anti-AD compound in the raw PN, and ginsenosides Rg_2_ and 20(*S*)-Rg_3_ might be the main anti-AD compounds in the steamed PN. The spectrum-effect relationship analyses revealed that the transformed saponins were partially responsible for the increased neuroprotective and antioxidant effects of steamed PN.

### 3.6. Verification Experiment

To further verify the reliability of the spectrum-effect relationship results, three identified constituents were inspected for their anti-A*β*_1-42_ and antioxidant effects, respectively. As shown in Supplementary Figure 2(a), notoginsenoside R_1_, ginsenosides Rg_2_, and 20*(S)*-Rg_3_ displayed neuroprotective effects as compared with the A*β*_1-42_-induced PC12 cell model group. All assay components showed inhibitory effect on peroxyl radical (Supplementary Figure 2(b)). The results were consistent with the predicted conclusion of the chemometric analysis.

## 4. Discussion

The processing methods of herbal medicines play an essential role in clinical applications of TCMs. Generally, the purpose of processing is to strengthen the curative efficacy, generate new effects, and reduce the toxicity or side-effects [32]. They involve special manipulations, such as steaming, baking, decocting, soaking, and so on [33]. PN is a medicinally important used species, and its roots have been used traditionally in both raw and processed forms. Raw PN is usually produced by air-drying, and processed PN is commonly made by a steaming process [34]. Steaming condition showed a great influence on the chemical constituents of PN. Based on our results, steaming process decreased the contents of original saponins presented in raw PN and increased the contents of several degradation compounds (Figure 2). The quantitative differences were correlated to the duration and temperature of steaming process. The result was consistent with the results from Wang et al. [35] and Xiong et al. [24]. The change of chemical composition and content can be extrapolated from the chemical structure. In the steaming condition, ginsenosides Rb_1_ and Rd are easy to selectively eliminate the sugar chain at C-20 to produce ginsenoside 20(*S*)*/*(*R*)-Rg_3_ and dehydrate ginsenoside Rg_3_ at C-20 to yield ginsenosides Rg_5_ and Rk_1_ [36]. Notoginsenoside R_1_ and ginsenosides Rg_1_ and Re are likely to first lose a glycosyl moiety at C-20 and subsequently its terminal sugar unit at C-6 to form ginsenosides Rg_2_ and/or Rh_1_. Ginsenoside Rh_1_ is further converted to Rk_3_ and Rh_4_ through dehydration at C-20 [21].

In addition to the chemical property, bioavailability and bioactivity of PN can be influenced significantly by steaming condition. The bioavailability of original saponins following oral administration of raw PN extract is relatively poor, which may be altered by intestinal microbiota to generate the less polar saponins [37]. These transformed less polar saponins are more easily absorbed and commonly exhibit much more potent activities, such as anticancer, antidiabetic, neuroprotective, and anti-inflammatory effects [38, 39]. Recently, saponins have garnered significant attention as a potential therapeutic agent in preventing and treating AD [40]. However, it is difficult to obtain higher quality saponin monomers for patient administration. Therefore, it is necessary to estimate the anti-AD activity of raw and steamed PN, which are two important types of clinical application. Based on our results, steamed PN extract had more potent antioxidant and neuroprotective properties compared to raw PN extract. Moreover, an increase in steaming duration and temperature significantly increased the anti-AD effect of PN at first, but a decreasing trend was subsequently observed (Figures 4 and 5). The decreased results might be due to the massive acrylamide was generated during the excessive thermal processing through Maillard reaction, which could cause neurotoxicity and create ROS overproduction [41]. The results indicated that the anti-AD activity of steamed PN samples is not fully related to the contents of less polar saponins.

Therefore, we optimized the steaming condition of PN for anti-AD study by combining chemical property, pharmacological property, and steaming duration. The impact of transformed saponin contents of PN on the change of anti-A*β*_1-42_ and antioxidant activities was shown in Supplementary Figure 3 and Supplementary Figure 4, respectively. At 105 and 110°C, these two activities enhanced as the transformed saponin contents increased. At 120°C, the samples exhibited stronger activity with shorter steaming time. After 4 h and 8 h steaming, the anti-A*β*_1-42_ and antioxidant effects reached their maximum with the increase of converted saponin contents, respectively. Therefore, the 120°C-4 h sample, which produced an abundant transformation of compositions and significant improvement of anti-A*β* and antioxidant properties with shorter steaming time, was considered as the optimized steaming process for the anti-AD test.

Chromatographic fingerprints and antioxidant and neuroprotective activities could differentiate the chemical characteristics and anti-AD properties of raw and steamed PN samples to a certain extent. However, it was still confused which constituents played a significant role in treatment. There is no doubt that the spectrum-effect relationship fits very well with the holistic mode of TCMs [42]. It develops integrated evaluation system and finally elucidates the active components in the fingerprint representing the curative effect [43]. In this study, PLS was preliminarily applied to predict potential anti-AD components of raw and steamed PN. According to the results, five characteristic peaks differently distributed in raw and steamed PN might be the potential active ingredients that respond to anti-AD. Three of them were identified as notoginsenoside R_1_ (P1), ginsenosides Rg_2_ (P6), and 20(*S*)-Rg_3_ (P15), respectively. The notoginsenoside R_1_ might be related to the anti-AD compounds in the raw PN, ginsenosides Rg_2_ and 20(*S*)-Rg_3_ might be related to the anti-AD compounds in the steamed form. Those ingredients could be the differential markers for the quality control of raw and steamed PN.

## 5. Conclusions

In this study, the approach which consisted in combining data from chemical characterization and neuroprotective/antioxidant activities of PN with different steaming parameters was constructed. The results indicated that steaming process showed a positive influence on the change of chemical constituent of raw PN as well as the activity of anti-A*β* and antioxidant effects. With the increase of steaming temperature and steaming time, the contents of original saponin (notoginsenoside R_1_, ginsenosides Rg_1_, Rb_1_, Re, and Rd) were decreased, while the transformed saponins (ginsenosides Rg_2_, Rh_1_, Rk_3_, Rh_4_, 20 (*S*)-Rg_3_, 20 (*R*)-Rg_3_, Rg_5_) were increased gradually. In the viewpoint of anti-AD activity on neuroprotective effect and antioxidant effect, the better results could be achieved in the shorter time at the higher temperature. By comparing with the steaming conditions, the steaming temperature in the steaming process had the greater impact on the change of the chemical composition and anti-AD activity of PN. Furthermore, the PLS analyses revealed that notoginsenoside R_1_ might be the main anti-AD compound in the raw PN, and ginsenosides Rg_2_ and 20(*S*)-Rg_3_ might be the main anti-AD compounds in the steamed PN. In conclusion, the steamed PN could be a useful resource for developing potential candidate targeting A*β* and oxidative stress against AD.

## Data Availability

The data used to support the findings of this study are available from the corresponding author upon request.

## Supplementary Materials

Supplementary Materials Supplementary data to this article are submitted together with the manuscript and are available online.
