# Supplementary material for: The Study of Steaming Durations and Temperatures on the Chemical Characterization, Neuroprotective, and Antioxidant Activities of Panax notoginseng
Source: Evid Based Complement Alternat Med. 2022 Jan 6;2022:3698518. doi: 10.1155/2022/3698518 (PMC8758266; doi:10.1155/2022/3698518)
Supplement: Supplementary Materials — Supplementary data to this article are submitted together with the manuscript and are available online. [file 3698518.f1.zip › Supplementary Materials/3698518.f1.docx]

**Supplementary Materials**

**The study of steaming durations and temperatures on the chemical characterization, neuroprotective and antioxidant activities of *Panax notoginseng***


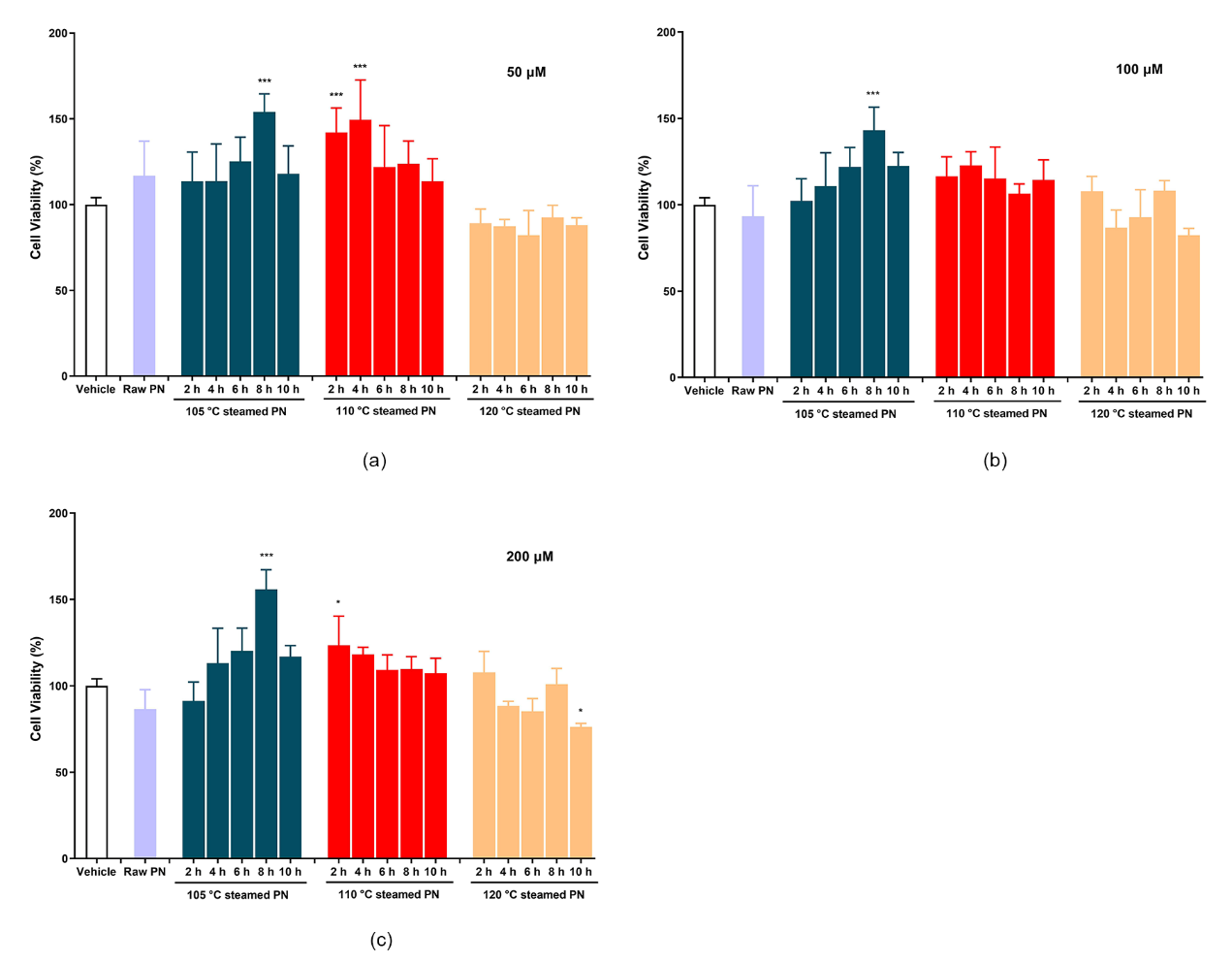


**Figure S1.** Cytotoxicity effects of raw and steamed *Panax notoginseng* (PN) at 50 μg/mL (A), 100 μg/mL (B) and 200 μg/mL (C) in PC12 cells. *^*^P<0.05*, *^**^P<0.01, ^***^P<0.005* versus vehicle control group were considered statistically significant differences.


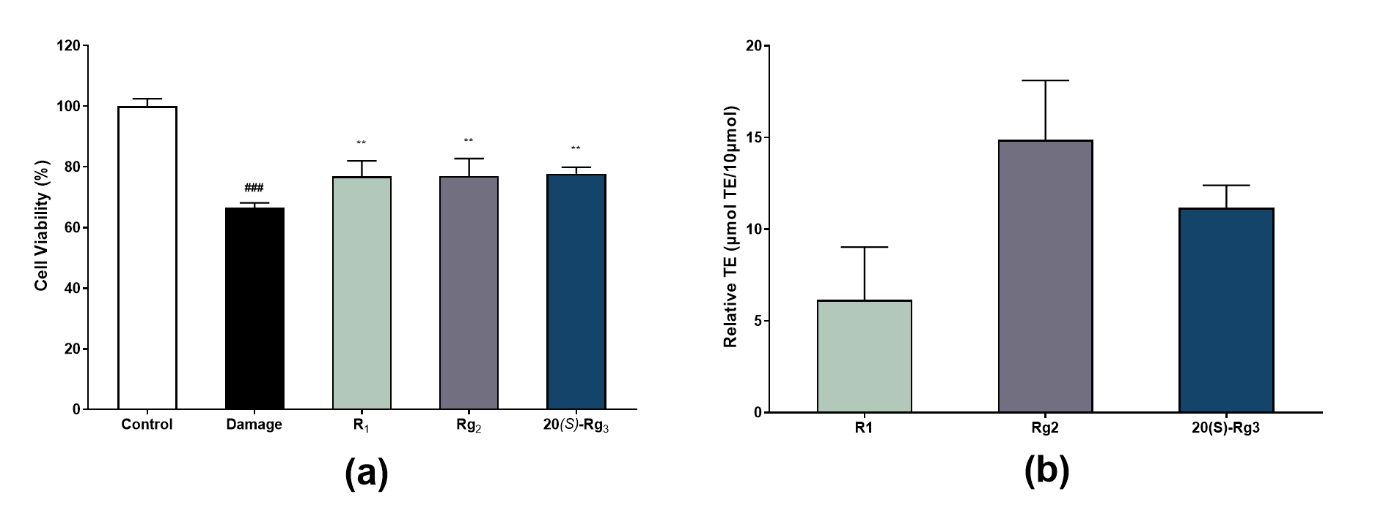


**Figure S2.** Anti-amyloid-*β* peptide effects in A*β*_1-42_-induced PC12 cell damage model (A) and oxygen radical absorbance capacity (ORAC) values (B) of three constituents from raw or steamed *Panax notoginseng*. *^###^P<0.005* versus the vehicle control group as indicated, *^*^P<0.05, ^**^P<0.01, ^***^P<0.005* versus A*β*_1-42_-induced model group were considered statistically significant differences.


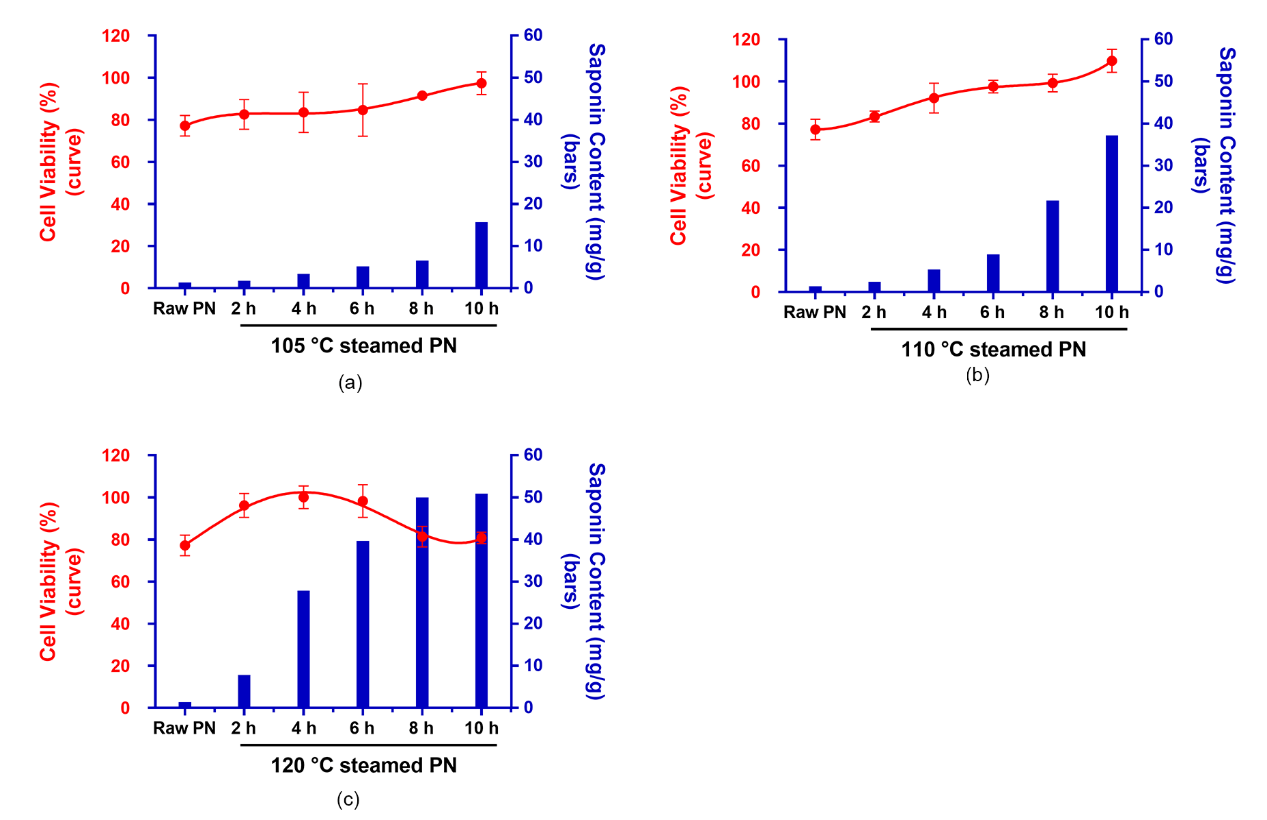


**Figure S3.** The impact of transformed saponin contents of raw and steamed *Panax notoginseng* (PN) on the anti-A*β*_1-42_ activity at 105 °C (A), 110 °C (B), 120 °C (C).


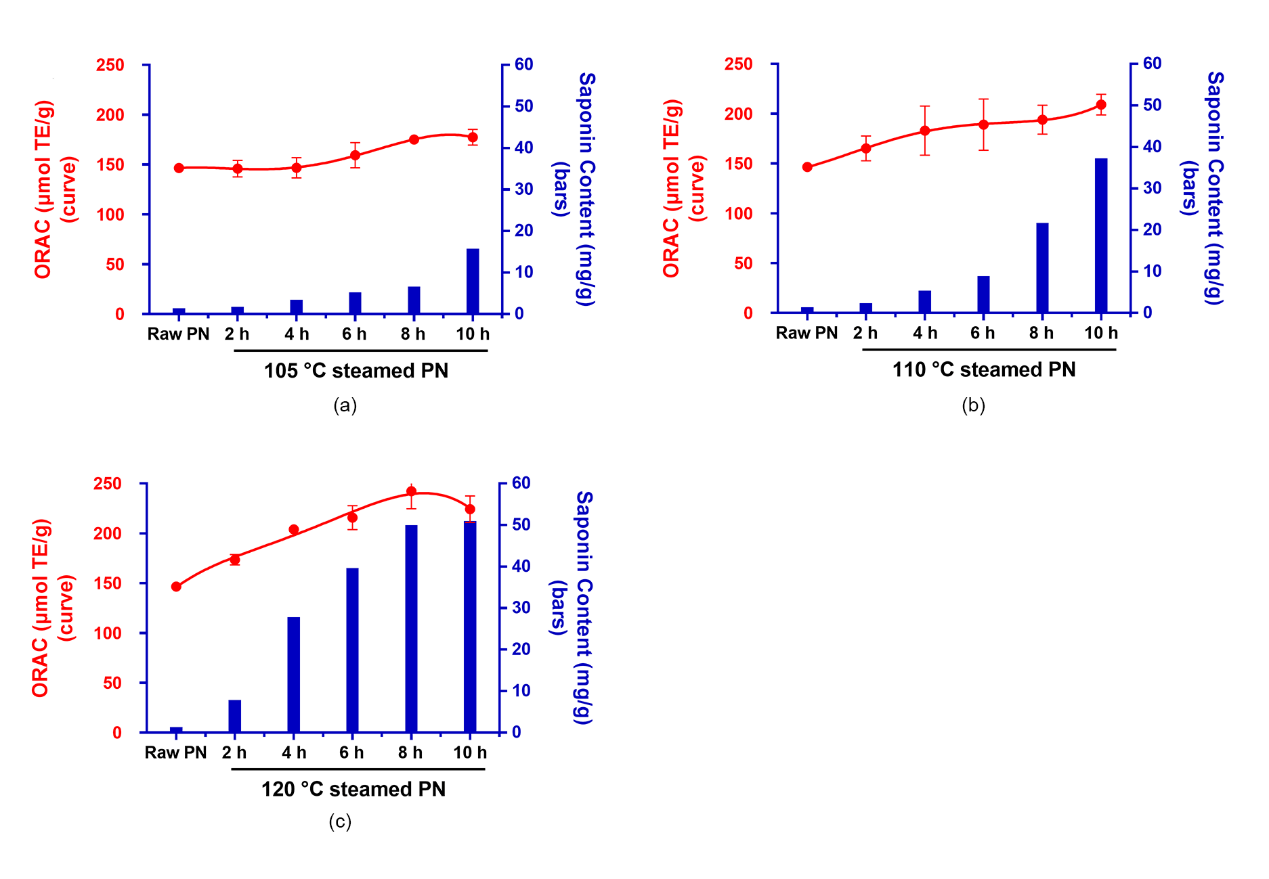


**Figure S4.** The impact of transformed saponin contents of raw and steamed *Panax notoginseng* (PN) on the oxygen radical absorbance capacity (ORAC) value at 105 °C (A), 110 °C (B), 120 °C (C).

Table S1. Linear regression data of twelve saponins in *Panax Notoginseng*

| **Analytes** | **Regression equation** | **R** | **Linear range (mg/mL)** |
| --- | --- | --- | --- |
| Notoginsenoside R_1_ | y = 4E+06x – 19204.0 | 0.9993 | 0.012-0.095 |
| Ginsenoside Rg_1_ | y = 4E+06x + 23538.0 | 0.9997 | 0.007-0.574 |
| Ginsenoside Re | y = 3E+06x + 2004.9 | 0.9998 | 0.008-0.078 |
| Ginsenoside Rb_1_ | y = 3E+06x + 13532.0 | 0.9998 | 0.012-0.466 |
| Ginsenoside Rg_2_ | y = 5E+06x + 3875.8 | 0.9995 | 0.006-0.042 |
| Ginsenoside Rh_1_ | y = 5E+06x + 8916.7 | 0.9997 | 0.008-0.180 |
| Ginsenoside Rd | y = 3E+06x - 502.4 | 0.9999 | 0.004-0.181 |
| Ginsenoside Rk_3_ | y = 1E+07x + 11802.0 | 0.9998 | 0.002-0.176 |
| Ginsenoside Rh_4_ | y = 8E+06x + 38004.0 | 0.9994 | 0.003-0.500 |
| Ginsenoside 20(*S*)- Rg_3_ | y = 4E+06x + 1673.0 | 0.9993 | 0.004-0.100 |
| Ginsenoside 20(*R*)- Rg_3_ | y = 5E+06x + 306.0 | 0.9995 | 0.006-0.045 |
| Ginsenoside Rg_5_ | y = 1E+07x + 20620.0 | 0.9995 | 0.010-0.163 |
